# Supplementary material for: Global hypo-methylation in a proportion of glioblastoma enriched for an astrocytic signature is associated with increased invasion and altered immune landscape
Source: eLife. 2022 Nov 22;11:e77335. doi: 10.7554/eLife.77335 (PMC9681209; doi:10.7554/eLife.77335)
Supplement: Figure 2—source data 1. [file elife-77335-fig2-data1.zip › Figure_2_source_data_1/Figure_2B/homerResults/motif9.similar.html]

motif9

## Information for motif9

A
C
G
T
A
G
T
C
A
G
T
C
A
C
G
T
A
C
G
T
A
C
G
T
A
G
T
C
A
C
G
T
A
C
T
G
A
G
T
C
A
C
G
T
A
C
G
T
  
Reverse Opposite:  

C
G
T
A
C
G
T
A
A
C
T
G
A
G
T
C
C
G
T
A
A
C
T
G
C
G
T
A
C
G
T
A
C
G
T
A
A
C
T
G
A
C
T
G
C
G
T
A
  

|  |  |
| --- | --- |
| p-value: | 1e-69 |
| log p-value: | -1.601e+02 |
| Information Content per bp: | 1.530 |
| Number of Target Sequences with motif | 118.0 |
| Percentage of Target Sequences with motif | 5.11% |
| Number of Background Sequences with motif | 2.9 |
| Percentage of Background Sequences with motif | 0.83% |
| Average Position of motif in Targets | 99.0 +/- 54.8bp |
| Average Position of motif in Background | 115.1 +/- 52.5bp |
| Strand Bias (log2 ratio + to - strand density) | 0.3 |
| Multiplicity (# of sites on avg that occur together) | 1.03 |
| Motif File: | file (matrix) reverse opposite |

### Similar de novo motifs found

|  |  |  |  |  |  |  |  |
| --- | --- | --- | --- | --- | --- | --- | --- |
| Rank | Match Score | Redundant Motif | P-value | log P-value | % of Targets | % of Background | Motif file |
| 1 | 0.913 | A C G T A G T C A G T C A C G T A C G T A C G T A G T C A C G T A C T G A G T C | 1e-49 | -113.512696 | 3.16% | 0.56% | motif file (matrix) |
| 2 | 0.617 | T C G A C T G A C T G A C T A G T C A G C T G A C T A G C T G A T C G A C T G A T G A C G T C A T C G A T C G A C T G A | 1e-24 | -57.254416 | 2.86% | 0.83% | motif file (matrix) |
